# Distinct clinical features and prognostic factors of hepatitis C virus-associated non-Hodgkin’s lymphoma: a systematic review and meta-analysis

**DOI:** 10.1186/s12935-021-02230-1

**Published:** 2021-10-09

**Authors:** Minyue Zhang, Fei Gao, Ling Peng, Lijing Shen, Peng Zhao, Beiwen Ni, Jian Hou, Honghui Huang

**Affiliations:** 1 Division of Hematology, Renji Hospital, School of Medicine, Shanghai Jiaotong University, Shanghai, 200127 China; 2 State Key Laboratory of Southwestern Chinese Medicine Resources, Pharmacy School, Chengdu University of Traditional Chinese Medicine, Chengdu, 611730 China; 3 Division of Chinese Medicine, M.D. Prefectural People’s Hospital, Chuxiong Yi Autonomous Prefecture, 675500 China

**Keywords:** Non-Hodgkin’s lymphoma, HCV infection, Prognosis, Antiviral treatment, Meta-analysis

## Abstract

**Background:**

Increasing evidence suggests that hepatitis C virus (HCV) infection is associated with non-Hodgkin’s lymphoma (NHL). However, no clear consensus has been reached about the clinical features and effective treatment of HCV-associated NHL patients. We therefore performed a systematic review and meta-analysis to explore the clinical characteristics and effectiveness of antiviral treatment or rituximab administration among NHL patients with HCV infection.

**Methods:**

Eight electronic databases, including PubMed, OVID, EMBASE, Cochrane Library, ClinicalTrials, WANFANG, CNKI, and VIP, were searched for eligible studies up to July 31, 2021. The hazard ratio (HR) or odds ratio (OR) corresponding to the 95% confidence interval (CI) was calculated to estimate the outcomes. Publication bias was assessed by Egger’s and Begg’s tests. Statistical analysis was performed with RevMan 5.4 software and Stata version 15.

**Results:**

There were 27 shortlisted articles out of a total of 13,368 NHL patients included in the current meta-analysis. Our results demonstrated that NHL patients with HCV infection had a significantly shorter overall survival (OS: HR 1.89; 95% CI 1.42–2.51, P < 0.0001) and progression-free survival (PFS: HR 1.58; 95% CI 1.26–1.98, P < 0.0001), a lower overall response rate (ORR: OR 0.58, 95% CI 0.46–0.73, P < 0.00001) and a higher incidence of hepatic dysfunction during chemotherapy (OR 5.96; 95% CI 2.61–13.62, P < 0.0001) than NHL patients without HCV infection. HCV-positive NHL patients exhibited an advanced disease stage, an elevated level of LDH, a high-intermediate and high IPI/FLIPI risk as well as a higher incidence of spleen and liver involvement. Moreover, antiviral treatment prolonged survival (OS: HR 0.38; 95% CI 0.24–0.60, P < 0.0001), reduced disease progression [PFS/DFS (disease-free survival): HR 0.63; 95% CI 0.46–0.86, P = 0.003] and reinforced the treatment response (ORR: OR 2.62; 95% CI 1.34–5.11, P = 0.005) among the HCV-infected NHL patients. Finally, rituximab administration was associated with a favourable OS, while liver cirrhosis and low levels of albumin predicted a poor OS for HCV-positive NHL patients.

**Conclusions:**

The current study provided compelling evidence about an inferior prognosis and distinct clinical characteristics among HCV-associated NHL patients. Antiviral treatment and rituximab-containing regimens were shown to be efficacious in improving the clinical outcomes of NHL patients with HCV infection.

**Supplementary Information:**

The online version contains supplementary material available at 10.1186/s12935-021-02230-1.

## Background

Non-Hodgkin’s lymphoma (NHL) is a group of lymphoid malignancies with high heterogeneity. It can be classified into B-cell, T-cell, and natural killer (NK)-cell lymphoma. Increasing amounts of evidence has shown that pathogen infection contributes to the pathogenesis of different subtypes of lymphoma, such as Epstein-Barr virus (EBV) in Hodgkin disease or Burkitt’s lymphoma, human T-cell leukaemia virus type 1 in adult T cell leukaemia and lymphoma, and *Helicobacter pylori* in gastric mucosa-associated lymphoid tissue [1–3]. According to the 2016 revision of the World Health Organization (WHO) classification of lymphoid neoplasms, two subtypes of NHL associated with specific viral infection, EBV-positive diffuse large B-cell lymphoma (DLBCL) and human herpesvirus type-8 (HHV-8)-positive DLBCL, have been classified as separate subtypes of DLBCL [4]. These new categories strongly support the hypothesis that NHL patients with specific infections display distinct clinical manifestations and prognoses.

Recently, hepatitis C virus (HCV) was found in peripheral blood mononuclear cells and lymph nodes [5, 6], and hepatitis C NS3 protein could be detected in tumour cells from patients with HCV-associated B-cell lymphoma [7], suggesting that HCV is also lymphotropic. In addition, a meta-analysis provided quantitative evidence that HCV infection could lead to a 2.5-fold increased risk of developing NHL [8]. Nevertheless, the clinical characteristics and prognosis of HCV-associated lymphoma are still undefined.

Increasing clinical attention has been focused on the association of HCV infection and the clinical outcomes of NHL patients. However, the results are still inconsistent based on the existing retrospective studies with small sample sizes [9–18]. Hosry et al. [17] found that HCV-associated DLBCL had unique clinical features and poor outcomes. However, other studies [14, 18] have shown that HCV infection is not associated with NHL patient survival. In addition, an increasing number of studies have explored the survival benefit of antiviral treatment or rituximab-containing chemotherapy in NHL patients with HCV infection, yet the results are controversial [15, 19–22]. In view of the limitations of previous studies, we performed a systematic review and meta-analysis aiming to evaluate the clinical features and prognostic factors of HCV-associated NHL patients. Moreover, the effect of antiviral treatment and rituximab administration on NHL patients with HCV infection was also investigated in the current study.

## Methods

### Identification of relevant studies

To identify all studies that explored the impact of HCV infection on the clinical outcomes of NHL patients or the effect of antiviral treatment and rituximab administration on NHL patients with HCV infection, literature searches of the PubMed, EMBASE, OVID, Cochrane Central Register of Controlled Trials (CENTRAL/CCTR) and ClinicalTrials (https://clinicaltrials.gov) databases were carried out up to July 31, 2021. The strategies of literature search were summarized in Additional file 1: Table S1. Additionally, literature searches were also performed in three Chinese databases, including WANFANG (http://www.wanfangdata.com), CNKI (https://www.cnki.net) and VIP (http://www.cqvip.com), with the same search strategies as those for English databases. References from the retrieved studies, meeting abstracts, relevant meta-analyses and systematic reviews were also screened. Case reports, editorials and review articles were excluded. When a publication overlapped with other publications of the same trial, only the article with more details or the most recent article was retained.

### Selection criteria and study outcomes

The studies included in the meta-analysis needed to satisfy all of the following criteria: (1) the study population was NHL patients. NHL was diagnosed by cytological examination or pathological examination according to the WHO Classification of Tumors of Hematopoietic and Lymphoid Tissues [4]; (2) HCV infection was defined as the detectable HCV-RNA or HCV antibody; and (3) The study focusing on the impact of HCV infection on NHL. The study population consisted of two groups: NHL patients with and without HCV infection. The clinical features or survivals between the two groups of patients were compared; or (4) The study focusing on the effect of antiviral or rituximab treatment on HCV-associated NHL. The study population consisted of two groups: HCV-infected NHL patients receiving and not receiving antiviral/rituximab treatment. The treatment response and clinical prognosis between the two groups of patients were analysed. The exclusion criteria were as follows: (1) patients co-infected with human immunodeficiency virus (HIV); (2) patients with post-transplant lymphoproliferative disorder; and (3) patient numbers less than or equal to ten in any study group. When the relevant data were not reported in the paper, we contacted the author to obtain the relevant information by e-mail or telephone.

The primary outcomes assessed the impact of HCV infection on the NHL patients’ prognoses, including overall survival (OS) and disease progression [progression-free survival (PFS)/disease-free survival (DFS)]. The other outcomes measured the treatment response [overall response rate (ORR)] and the clinical characteristics of NHL patients with HCV infection, including the age of disease onset (< 60 years old); the presence of B symptoms; advanced disease stage (Ann Arbor staging III/IV); involvement of the spleen, liver and bone marrow; elevated LDH level; high-intermediate and high IPI/FLIPI risk; and the incidence of hepatic dysfunction during chemotherapy. Furthermore, the effect of antiviral therapy or rituximab administration as well as liver-related prognostic factors for HCV-positive NHL patients was also investigated. Since PFS and DFS are similar outcomes reflecting disease progression, we pooled PFS and DFS for analysis in the current study.

### Data extraction

Two reviewers (Gao F and Peng L) independently extracted the data and outcomes using an electronic standardized form. The following information from each study was summarized: (1) first author, (2) year of publication, (3) country, (4) subtypes of NHL, (5) number of patients with and without HCV infection, (6) number of HCV-positive NHL patients receiving and not receiving antiviral/rituximab treatment, (7) anti-lymphoma therapeutic regimens, (8) antivirus treatment regimens, and (9) patient characteristics. Any discrepancies between the two reviewers were resolved by an additional investigator, Zhang M.

### Quality assessment

The Newcastle–Ottawa Quality Assessment Scale (NOS) was adopted to assess the methodological quality of the included studies [23]. The following three items were evaluated: (1) patient selection, (2) comparability of the intervention and observation groups, and (3) assessments of the outcomes.

### Statistical analysis

The hazard ratio (HR) corresponding to the 95% confidence interval (CI) was used to assess OS and PFS/DFS. If both the univariate and multivariate analysis results were reported in the included study, the latter was used in the meta-analysis. If the HRs and 95% CIs were not available from the original article, Kaplan–Meier curves of the included studies were reanalysed by Engauge digitizer software. The HRs and 95% CIs were indirectly calculated from the Kaplan–Meier curves using Tierney’s methods [24]. Odds ratios (ORs) corresponding to the 95% CIs were calculated to estimate the other outcomes. Publication bias was assessed by Egger and Begg’s tests. When the outcome was assessed by fewer than five included studies, publication bias tests were not performed. The methods of the meta-analyses and publication bias tests have been reported in our previous publications [25–28]. Statistical analysis was performed with ReviewManager 5.4 software (The Cochrane Collaboration, Oxford, UK) and Stata version 15 (Stata Corp, College Station, Texas, USA). All P-values were two-sided, and a P-value of < 0.05 was considered significant.

## Results

### Characteristics of the eligible studies

**Fig. 1:**
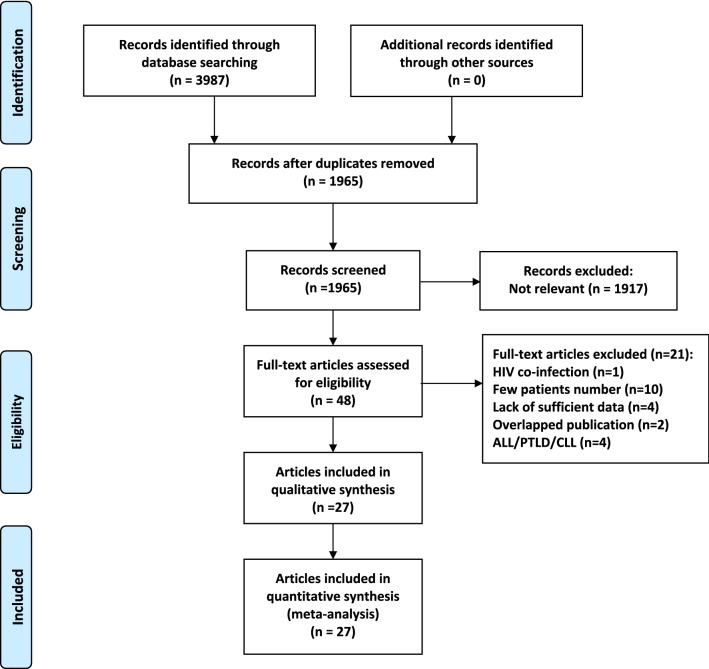
Literature search and selection. (**Abbreviation:** *ALL* acute lymphoblastic leukemia, *CLL* chronic lymphocytic leukemia, *PTLD* posttransplant lymphoproliferative disorders)

After the comprehensive literature search, 3987 articles were identified as potentially relevant publications. Upon further evaluation of the full text, 21 articles were excluded. Thus, 27 articles (29 studies) [9–22, 29–41] with a total of 13,368 NHL patients, including 3063 patients with HCV infection and 10,305 patients without HCV infection, fulfilled the inclusion criteria and were included in the current meta‐analysis (Fig. 1). The characteristics of the included articles are summarized in Table 1. The articles were published between 1997 and 2020 with sample sizes ranging from 58 to 5586. The majority of the studies were conducted in Italy (n = 11), Japan (n = 4), Egypt (n = 3), China (n = 2), France (n = 2) and the United States (n = 2). The other articles consisted of a single study each conducted in Russia, Korea, and Spain. Among the included articles, 14 articles focused on DLBCL, two articles on follicular lymphoma and two articles on marginal zone lymphoma. In addition to the aforementioned lymphoma subtypes, the patients in 9 articles had more than two subtypes of NHL. With regard to the methodological quality, most of the included articles had reliable quality, as indicated by NOS scores ≥ 6 points, except for 2 articles [30, 38] with NOS scores of 5 points (Additional file 2: Table S2).

**Table 1:** Baseline characteristics of included studies

Author	year	Country	Diagnosis	Regimes of anti-lymphoma treatment	Regimes of AT treatment	Follow-up (m) Median (range)	HCV infection		R use in HCV + patients		AT in HCV + patients		Hazard ratio
							yes	no	yes	no	yes	no	
Besson [9]	2006	France	DLBCL	–	–	47 (0–112)	26	5560	–	–	–	–	Extrapolated
Chen [10]	2015	China	DLBCL	I-CHT	–	36 (0.84–96.24)	29	139	29	0	–	–	Extrapolated
Chen [11]	2015	China	DLBCL	I-CHT	–	–	22	102	15	7	–	–	Reported
Dlouhy [12]	2017	Spain	DLBCL	I-CHT	–	48 (1.2–146.4)	31	290	31	0	–	–	Extrapolated
Elbedewy [13]	2020	Egypt	DLBCL	I-CHT	–	–	76	277	33	43	34	42	Reported
Ennishi [14]	2010	Japan	DLBCL	I-CHT	DAA	HCV+ 31 (4–42) HCV− 32 (5–51)	131	422	131	0	7	124	Reported
Hosry [17]	2016	USA	DLBCL	I-CHT	IFN, Rib, DAA	> 6	76	228	76	0	26	40	Extrapolated
Nishikawa [29]	2012	Japan	DLBCL	I-CHT	–	HCV+ 40.8 (3.6–92.4) HCV− 31.2 (2.4–94.8)	28	220	28	0	0	28	Extrapolated
Park [16]	2008	Korea	DLBCL	I-CHT	–	37.8	32	371	–	–	–	–	Extrapolated
Shimono [18]	2019	Japan	ps-DLBCL	I-CHT	–	HCV + 46.9 (4.0–94.3) HCV− 36.8 (0.8–133.5)	12	46	10	2-	–	–	Extrapolated
			Ordinary DLBCL	I-CHT	–	HCV+ 27.8 (1.9–122.1) HCV− 35.9 (0.1–90.1)	25	120	–	–	–	–	Extrapolated
Arcaini [30]	2007	Italy	Non-gastric MALToma	CHT, RT, surgery	–	–	60	112	–	–	–	–	Extrapolated
Arcaini [31]	2006	Italy	SMZL	splenectomy, IFN	–	37.2	49	206	–	–	–	–	–
Hosry [32]	2020	USA	FL	I-CHT, RT	IFN, Rib, DAA	–	19	57	–	–	13	6	–
Nesterova [33]	2020	Russia	FL	I-CHT	DAA	–	11	105	11	0	11	0	–
De Vita [34]	1997	Italy	B-NHL	–	–	–	35	122	–	–	–	–	–
Luppi [35]	1998	Italy	B-NHL	IFN, CHT	–	72	35	122	0	35	–	–	Extrapolated
La Mura [36]	2008	Italy	NHL	CHT	IFN, Rib	–	69	274	0	69	25	44	Extrapolated
Strianese [37]	2010	Italy	OA lymphoma	I-CHT, RT	–	Mean 75 (12–185)	23	106	–	–	–	–	Extrapolated
Tajima [38]	2017	Japan	NHL	–	–	–	66	1316	–	–	–	–	–
Vallisa [39]	1999	Italy	NHL	CHT, surgery	–	30 (1–68)	65	110	0	65	–	–	–
Arcaini [19]	2014	Italy	indolent NHL	surgery, I-CHT, RT	IFN, Rib	42 (6–204)	704	0	126	578	134	570	Reported
Merli [15]	2014	Italy	DLBCL	surgery, I-CHT, RT	IFN, Rib	24 (12–168)	535	0	255	280	23	512	Reported
Michot [20]	2015	France	NHL	surgery, I-CHT, RT	IFN, Rib	31 (19–71)	116	0	–	–	70	46	Reported
Persico [40]	2018	Italy	DLBCL	I-CHT	DAA	12	121	0	70	51	20	101	Extrapolated
Rattotti [21]	2019	Italy	Indolent NHL	I-CHT, antibiotics	IFN, Rib	36 (1‐84)	138	0	–	–	36	102	Extrapolated
			Aggressive NHL				112	0	–	–	17	95	
Salah-Eldin [41]	2014	Egypt	DLBCL	I-CHT	–	41 (38.6–43.4)	280	0	200	80	0	0	Reported
Zaky [22]	2014	Egypt	DLBCL	I-CHT	–	36 (5–42)	137	0	68	69	–	–	Extrapolated

**Abbreviation:** *AT* antiviral treatment, *CHT* chemotherapy, *DAA* direct-acting antiviral, *DLBCL* diffuse large B-cell lymphoma, *FL* follicular lymphoma, *I-CHT* immunochemotherapy, *IFN* interferon, *MALToma* mucosa-associated lymphoid tissue lymphoma, *NHL* non-Hodgkin’s lymphoma, *OA* ocular adnexal, *ps-DLBCL* primary splenic diffuse large B-cell lymphoma, *SMZL* splenic marginal zone lymphoma, *R* rituximab, *RT* radiotherapy, *Rib* Ribavirin

### The impact of HCV infection on NHL patients’ prognosis

**Fig. 2:**
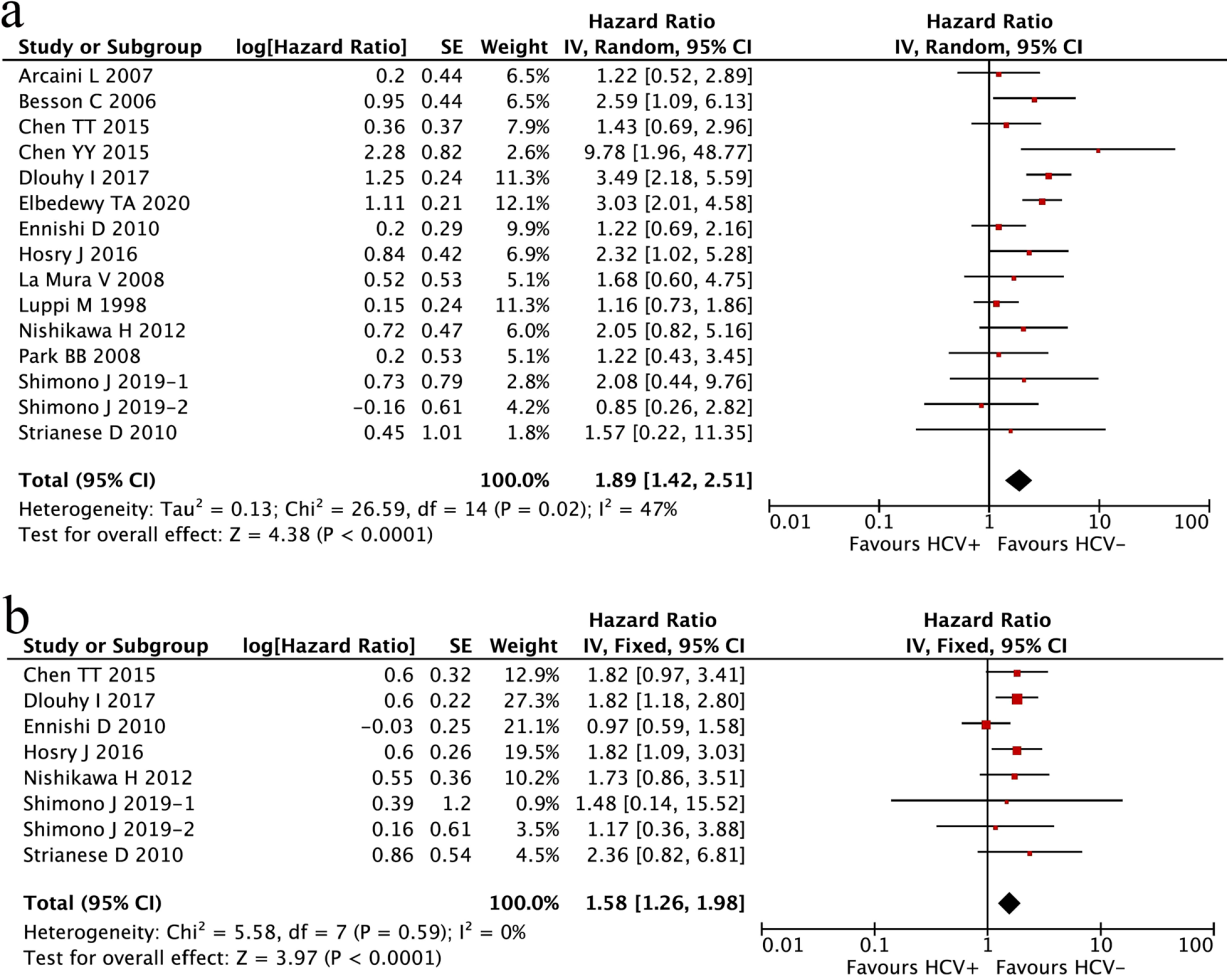
Meta-analysis of the association between the status of HCV and the prognosis of NHL patients. **a** Overall survival (OS);** b** progression-free survival (PFS)

Among all 29 studies, 15 studies were available for the analyses of OS. Due to obvious heterogeneity among these studies (I^2^ = 47%, P_heterogeneity_ = 0.02), a random-effects model was used to pool all HRs and their 95% CIs. Meta-analysis revealed that HCV-positive patients had significantly worse OS than HCV-negative patients (HR 1.89; 95% CI 1.42–2.51, P < 0.0001; Fig. 2a).

A total of 8 studies were eligible for the assessment of PFS. A fixed-effects model was used to calculate the results, as there was no heterogeneity among the included studies (I^2^ = 0%, P_heterogeneity_ = 0.59). The results demonstrated that patients with HCV infection showed a significantly shortened PFS compared with patients without HCV infection (HR 1.58; 95% CI 1.26–1.98, P < 0.0001; Fig. 2b).

### HCV infection and treatment response

**Fig. 3:**
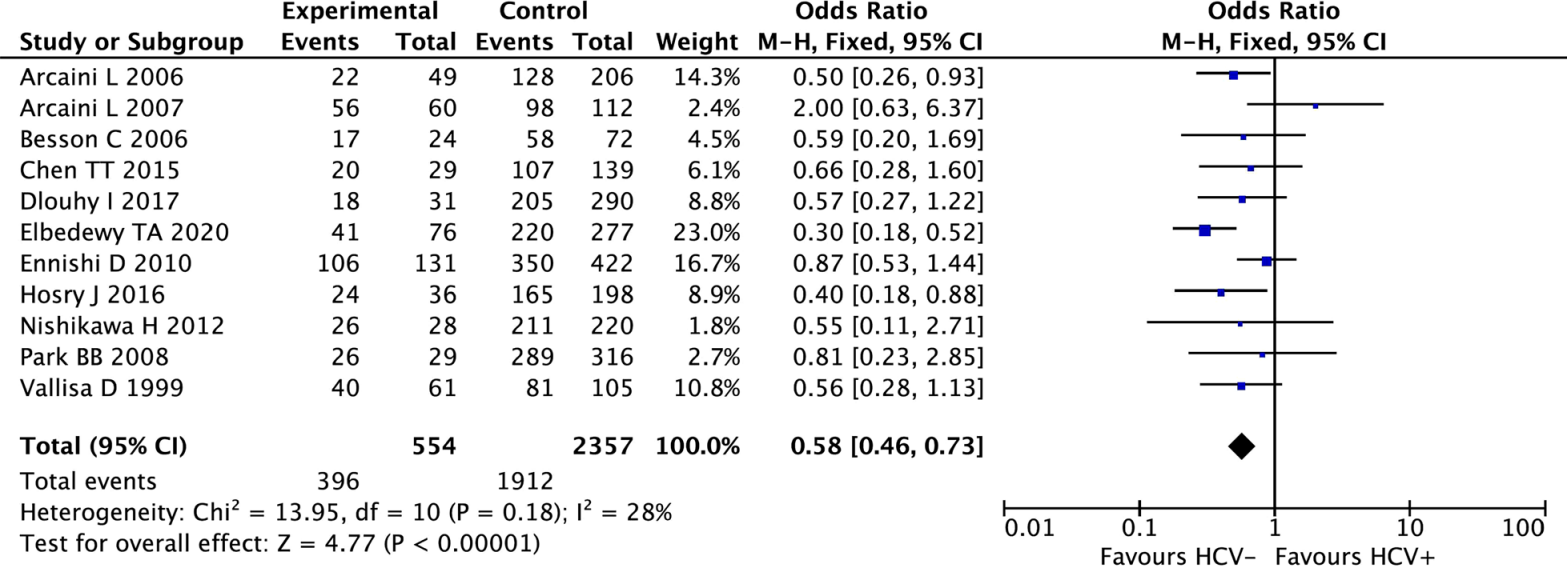
Meta-analysis of the association between HCV status and the overall response rate (ORR) of NHL patients

Eleven studies were identified to assess the ORR between NHL patients with and without HCV infection. The OR and 95% CI of the ORR were pooled by using a fixed-effects model, as the heterogeneity tests suggested no significant heterogeneity (I^2^ = 28%, P_heterogeneity_ = 0.18). The combined results showed that the ORR of HCV-positive NHL patients was significantly lower than that of HCV-negative NHL patients (OR 0.58, 95% CI 0.46–0.73, P < 0.00001, Fig. 3).

### HCV infection and hepatic dysfunction during chemotherapy

**Fig. 4:**
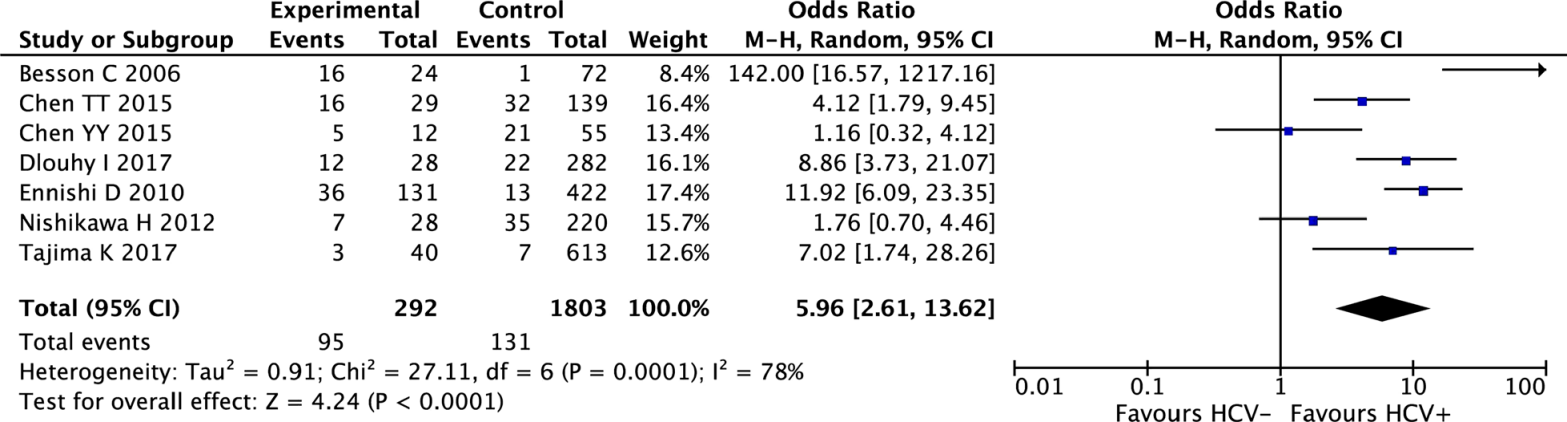
Meta-analysis of the association between HCV status and hepatic dysfunction of NHL patients during chemotherapy

In total, 7 studies including 2095 participants (DLBCL patients) were eligible for the analysis of this outcome by the random-effects model (I^2^ = 78%, P_heterogeneity_ = 0.0001). As shown in Fig. 4, a higher incidence of hepatic dysfunction during chemotherapy was observed in HCV-infected patients than in HCV-noninfected patients (OR 5.96; 95% CI 2.61–13.62, P < 0.0001).

### HCV infection and clinical characteristics

The clinical features of NHL patients were also comprehensively compared between HCV-positive and HCV-negative NHL patients. The results indicated that HCV infection was associated with an advanced disease stage (OR 1.42, 95% CI 1.14–1.76, P = 0.001, Additional file 3: Figure S1), an elevated level of LDH (OR 1.44; 95% CI 1.17–1.79, P = 0.0008, Additional file 4: Figure S2), a high-intermediate and high IPI/FLIPI risk (OR 1.29; 95% CI 1.07–1.56, P = 0.008, Additional file 5: Figure S3), as well as a higher incidence of spleen involvement (OR 2.95; 95% CI 2.17–4.02, P < 0.00001, Additional file 6: Figure S4) and liver involvement (OR 2.01; 95% CI 1.46–2.78, P < 0.0001, Additional file 7: Figure S5). However, there was no significant difference in the age of disease onset (Additional file 8: Figure S6), presence of B symptoms (Additional file 9: Figure S7) or the incidence of the involvement of bone marrow (Additional file 10: Figure S8) between the two groups of patients.

### Effect of antiviral treatment and rituximab administration on HCV-associated NHL patients

**Fig. 5:**
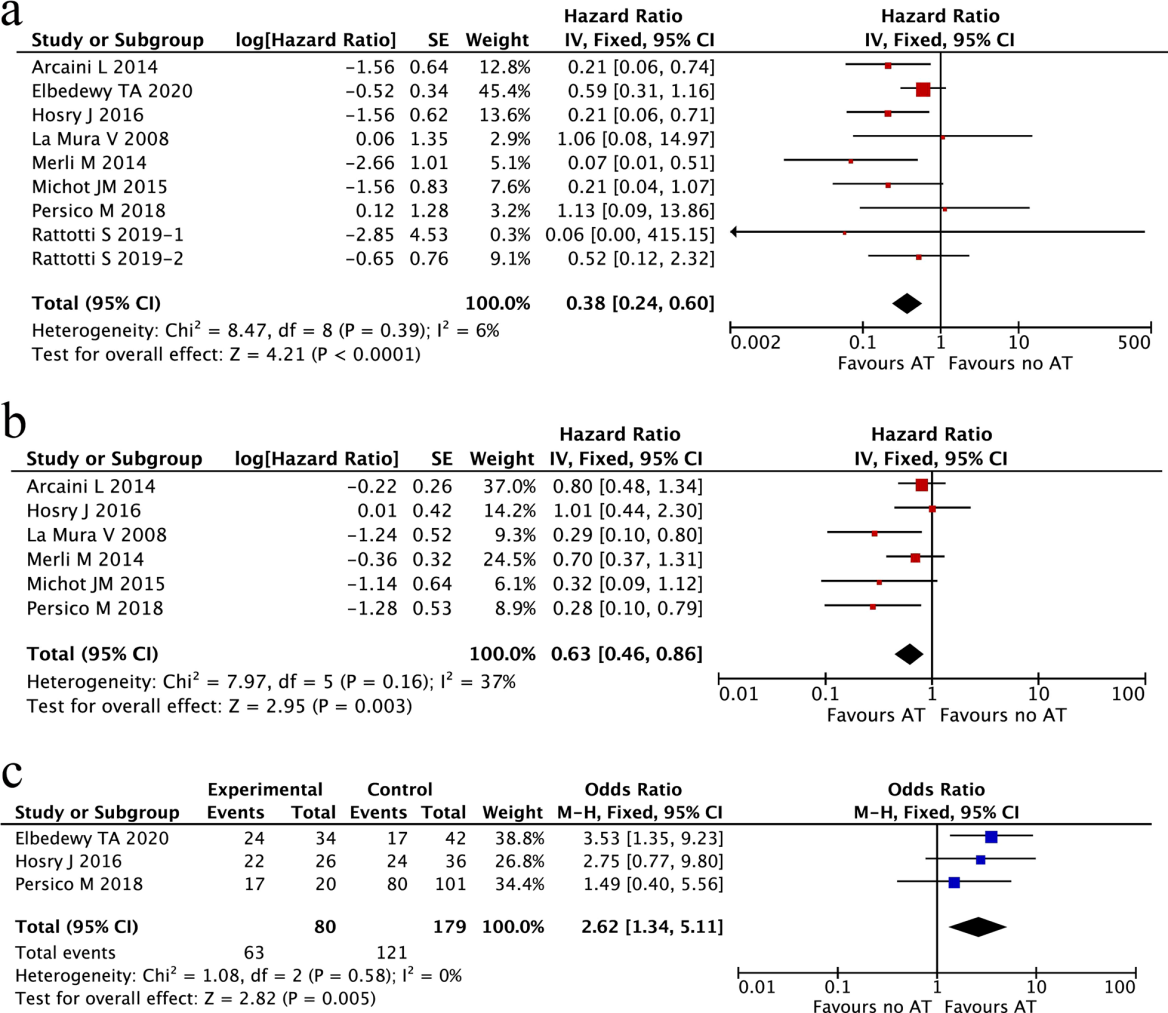
Meta-analysis of the clinical outcomes between HCV-associated NHL patients receiving antiviral treatment and those not receiving antiviral treatment. **a** Overall survival (OS); **b** progression-free survival/disease-free survival (PFS/DFS); **c** overall response rate (ORR)

Since NHL patients with HCV infection were found to have disadvantages of survival and treatment responses, whether antiviral treatment may improve the clinical outcomes of HCV-associated NHL patients was also investigated. Among all of the studies reviewed, 9 studies including 1838 HCV-infected NHL patients were included to evaluate the impact of antiviral treatment on OS, PFS/DFS and ORR. As shown in Fig. 5, antiviral treatment was associated with an improved OS (HR 0.38; 95% CI 0.24–0.60, P < 0.0001; Fig. 5a) and PFS/DFS (HR 0.63; 95% CI 0.46–0.86, P = 0.003; Fig. 5b), as well as a higher ORR (OR 2.62; 95% CI 1.34–5.11, P = 0.005, Fig. 5c) in comparison with patients without antiviral treatment.

**Fig. 6:**
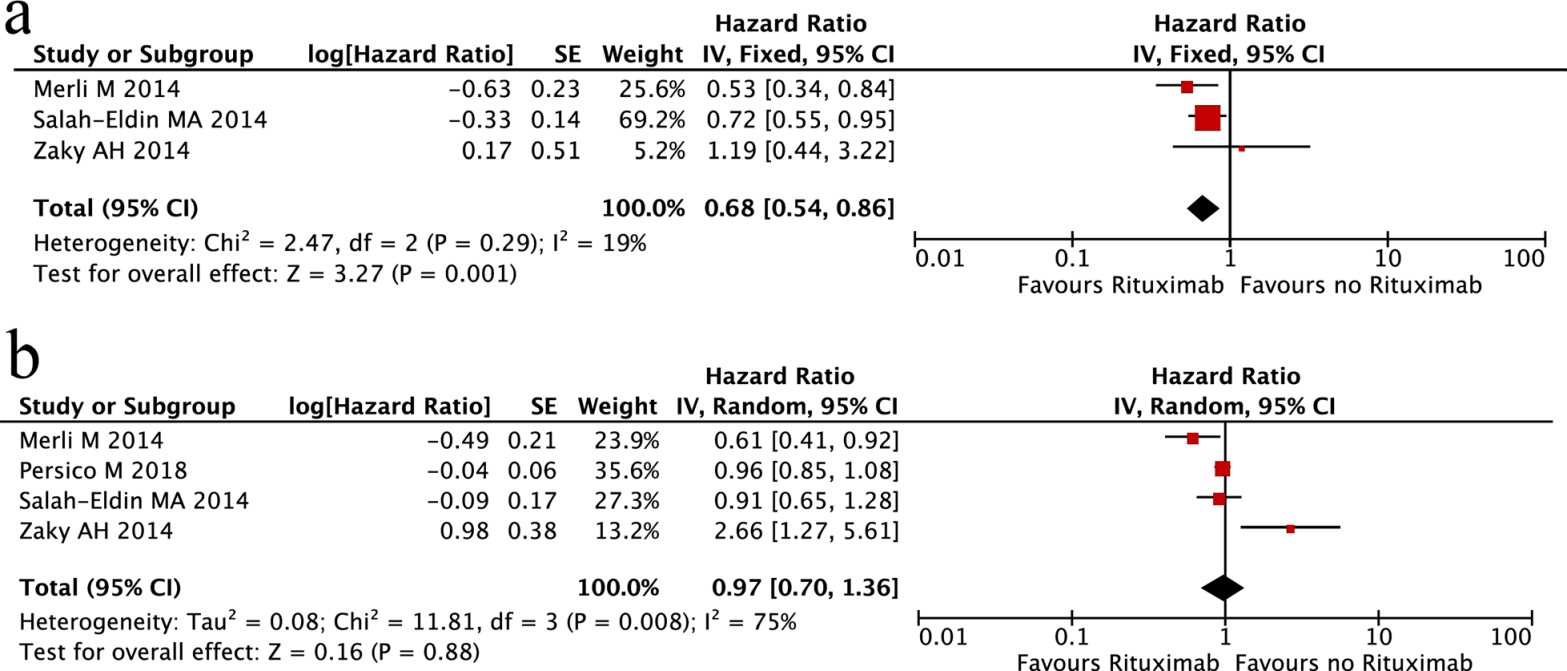
Meta-analysis of clinical outcomes between HCV-associated NHL patients receiving rituximab-containing chemotherapy and rituximab-free chemotherapy. **a** Overall survival (OS); **b** progression-free survival (PFS)

In addition, we also explored whether rituximab-containing regimens could improve the clinical prognosis of HCV-positive NHL patients. Four studies comprising 688 participants (DLBCL patients) were evaluated. The results demonstrated that patients treated with rituximab-containing chemotherapy exhibited favourable OS compared with those receiving rituximab-free chemotherapy (HR 0.68; 95% CI 0.54–0.86, P = 0.001; Fig. 6a). However, no difference in PFS was observed between these two groups of patients (HR 0.97; 95% CI 0.70–1.36, P = 0.88; Fig. 6b).

### Liver-related survival factors in HCV-associated NHL patients

**Fig. 7:**
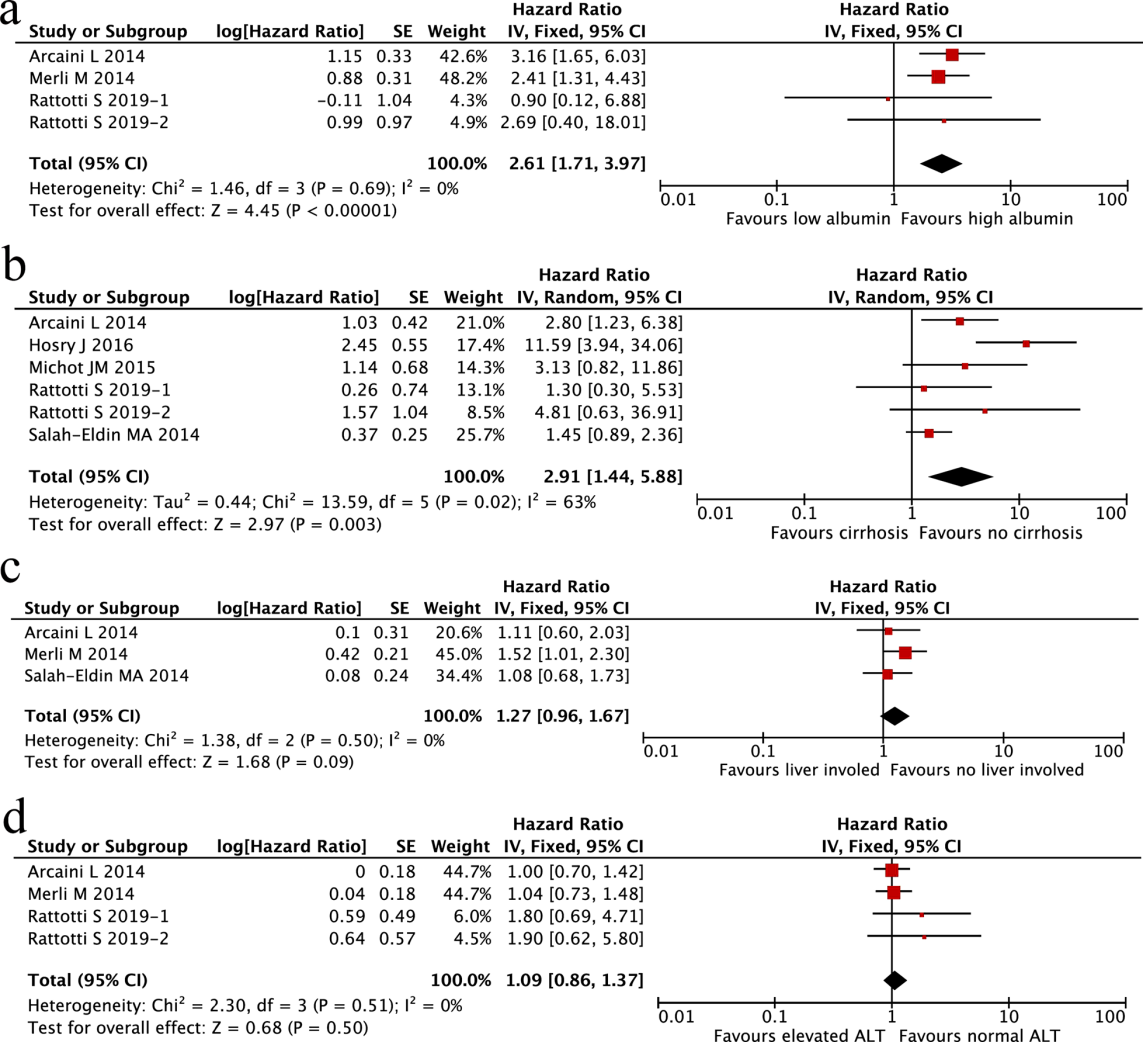
Meta-analysis of prognostic factors among HCV-associated NHL patients. **a** Liver cirrhosis; **b** low level of albumin; **c** liver involvement; **d** elevated alanine transaminase (ALT)

We subsequently evaluated liver-related survival factors in HCV-associated NHL patients, including liver cirrhosis, liver involvement, low levels of albumin (< 3.5 g/dl) and elevated alanine transaminase (ALT). As shown in Fig. 7a–d, low levels of albumin (HR 2.61; 95% CI 1.71–3.97, P < 0.00001; Fig. 7a) and liver cirrhosis (HR 2.91; 95% CI 1.44–5.88, P = 0.003; Fig. 7b) were significantly associated with an inferior OS. There was a trend for an association between liver involvement and a short OS (HR 1.27; 95% CI 0.96–1.67, P = 0.09; Fig. 7c). However, no association was found between elevated ALT and OS (HR 1.09; 95% CI 0.86–1.37, P = 0.50; Fig. 7d).

### Subgroup analysis

Subgroup analysis was conducted based on the prevalence of HCV infection. According to the 2017 report of the prevalence of HCV infection from the WHO [42], the countries among the included studies were divided into high prevalence of HCV infection, including Italy, Spain, France, Russia and Egypt, and low prevalence of HCV infection, including the United States, Japan, Korea, and China (using a cut-off value of HCV infection prevalence at 1.5%). The results of the subgroup analysis are shown in Table 2 (high prevalence) and Table 3 (low prevalence). Compared with the results from the overall population, similar results could be observed in the subgroup analysis. NHL patients with HCV infection had an inferior prognosis, a reduced treatment response and a higher incidence of hepatic dysfunction in both subpopulations. With regard to clinical features, HCV infection had no effect on the IPI/FLIPI risk in either subgroup of patients, which was inconsistent with this outcome in the overall population. In addition, HCV-associated NHL patients had an early age of disease onset among the countries with a low prevalence of HCV infection. For the outcomes of the effect of antiviral and rituximab treatment as well as survival factors in HCV-positive NHL patients, all of the included studies were performed in countries with a high prevalence of HCV infection, except one study [17]. Therefore, subgroup analysis was not performed.

**Table 2:** Subgroup analysis in countries with high prevalence of HCV infection

Outcome	Studies	HR/OR (95%CI)	P value	Heterogeneity	
				I^2^%	P value
Impact of HCV infection					
Overall survival	7	2.07 (1.37–3.14)	0.0006	61	0.02
Progression-free survival	2	1.89 (1.27–2.82)	0.002	0	0.66
Overall response rate	6	0.51 (0.38–0.68)	< 0.00001	46	0.10
Hepatic dysfunction	2	30.13 (1.75–517.77)	0.02	84	0.01
Age of onset	5	1.04 (0.50–2.16)	0.92	79	0.0009
Advanced disease stage	11	1.53 (1.22–1.91)	0.0002	44	0.06
Presence of B symptom	6	0.99 (0.53–1.84)	0.97	63	0.02
Elevated LDH level	5	1.33 (0.96–1.84)	0.09	49	0.10
H-I/H risk	6	1.53 (0.96–2.44)	0.07	60	0.03
Spleen involvement	4	3.74 (2.32–6.02)	< 0.00001	0	0.52
Liver involvement	5	1.98 (1.23–3.20)	0.005	41	0.15
Bone marrow involvement	6	1.39 (0.97–2.00)	0.07	46	0.10

**Abbreviation:*** H-I/H* high-intermediate and high

**Table 3:** Subgroup analysis in countries with low prevalence of HCV infection

Outcome	Studies	HR/OR (95%CI)	P value	Heterogeneity	
				I^2^%	P value
Impact of HCV infection					
Overall survival	8	1.59 (1.16–2.17)	0.004	16	0.31
Progression-free survival	6	1.45 (1.10–1.90)	0.008	0	0.52
Overall response rate	5	0.70 (0.49–1.00)	0.05	0	0.59
Hepatic dysfunction	5	3.87 (1.60–9.36)	0.003	76	0.002
Age of onset	4	1.97 (1.18–3.28)	0.01	4	0.37
Advanced disease stage	10	1.22 (0.98–1.52)	0.07	28	0.18
Presence of B symptom	4	0.80 (0.53–1.21)	0.30	47	0.13
Elevated LDH level	6	1.54 (1.16–2.04)	0.003	26	0.24
H-I/H risk	8	1.16 (0.89–1.51)	0.26	0	0.52
Spleen involvement	5	2.48 (1.65–3.74)	< 0.0001	0	0.63
Liver involvement	7	2.04 (1.32–3.15)	0.001	17	0.30
Bone marrow involvement	8	0.98 (0.72–1.34)	0.90	0	0.46

**Abbreviation:*** H-I/H* high-intermediate and high

Since NHL is a group of heterogeneous diseases, we performed subgroup analysis based on the pathological types of NHL. In the current study, the majority of the included studies focused on DLBCL; hence, subgroup analysis was conducted in DLBCL patients. Similar to the overall patients, DLBCL patients with HCV infection had shorter OS and PFS, lower ORR and distinct clinical features in comparison to DLBCL patients without HCV infection. Moreover, HCV-associated DLBCL patients receiving antiviral treatment shared a better OS and PFS/DFS than patients without antiviral treatment. Due to the small sample size, subgroup analysis was not performed in terms of the outcomes of liver-related survival factors. The results of the subgroup analysis of DLBCL patients are summarized in Table 4.

**Table 4:** Subgroup analysis in DLBCL patients

Outcome	Studies	HR/OR (95%CI)	P value	Heterogeneity	
				I^2^%	P value
Impact of HCV infection					
Overall survival	11	2.29 (1.85–2.84)	< 0.00001	46	0.05
Progression-free survival	7	1.55 (1.23–1.95)	0.0002	0	0.54
Overall response rate	8	0.55 (0.42–0.72)	< 0.00001	23	0.24
Hepatic dysfunction	6	5.89 (2.31–15.01)	0.0002	82	< 0.0001
Age of onset	7	1.31 (0.67–2.57)	0.43	70	0.003
Advanced disease stage	11	1.20 (0.98–1.48)	0.08	14	0.31
Presence of B symptom	6	1.16 (0.70–1.94)	0.56	59	0.03
Elevated LDH level	9	1.47 (1.17–1.85)	0.0009	46	0.06
H-I/H risk	10	1.34 (1.07–1.67)	0.01	0	0.48
Spleen involvement	5	2.75 (1.92–3.95)	< 0.00001	0	0.55
Liver involvement	7	1.87 (1.22–2.85)	0.004	0	0.60
Bone marrow involvement	6	1.03 (0.71–1.50)	0.86	17	0.31
Impact of anti-viral treatment					
Overall survival	5	0.40 (0.24–0.66)	0.0003	37	0.18
Progression-free survival/disease-free survival	4	0.60 (0.39–0.92)	0.02	41	0.17

**Abbreviation:*** H-I/H* high-intermediate and high

In terms of antiviral treatment, subgroup analysis was performed based on the regimens of antiviral treatment. Among the 9 included studies, the patients in most studies (n = 6) received interferon (IFN) ± ribavirin as antiviral treatment. Only two studies [13, 40] focused on new direct-acting antiviral (DAA) treatments of HCV, and another study [17] consisted of different types of antiviral treatment. Therefore, subgroup analysis was conducted in patients receiving IFN ± ribavirin. The results suggested that patients receiving IFN ± ribavirin had obviously improved OS (HR 0.25; 95% CI 0.12–0.52, P = 0.0002, figure not shown) and PFS (HR 0.63; 95% CI 0.44–0.90, P = 0.01, figure not shown) compared with patients without antiviral treatment.

### Sensitivity analysis

Sensitivity analysis was performed by sequentially excluding individual studies to determine the origin of the heterogeneity and to verify the sensitivity of the results. The origin of the heterogeneity and overall effect after removal of the origin of the heterogeneity for each outcome are summarized in Table 5. The results showed that removal of the origin of the heterogeneity did not affect the effect size for each outcome, suggesting the stability of the results of the meta-analysis. However, for the outcomes of age of disease onset and hepatic dysfunction during chemotherapy, heterogeneity still existed even though the studies were excluded one by one.

**Table 5:** Results of sensitivity analysis

Outcome	Origin of heterogeneity	Overall effect	P value
Overall survival	Dlouhy I 2017 [12]	1.78 (1.45–2.18)	< 0.00001
Advanced disease stage	Vallisa D 1999 [39]	1.28 (1.09–1.51)	0.003
Presence of B symptoms	Arcaini L 2006 [31]	1.09 (0.84–1.41)	0.51
Liver cirrhosis	Hosry J 2016 [17]	1.84 (1.26–2.69)	0.002

### Publication bias

Begg’s test and Egger’s test were performed to assess publication bias. As shown in Table 6, no significant publication bias was observed for any of the outcomes.

**Table 6:** Results of Begg’s test and Egger’s test

Outcome	P _Begg’s test_	P _Egger’s test_
Impact of HCV infection		
Overall survival	1.000	0.619
Progression-free survival	0.386	0.871
Overall response rate	0.276	0.373
Hepatic dysfunction	0.452	0.236
Age of onset	1.000	0.499
Advanced disease stage	0.162	0.468
Presence of B symptom	0.074	0.065
Elevated LDH level	0.533	0.639
H-I/H risk	0.125	0.379
Spleen involvement	0.386	0.878
Liver involvement	1.000	0.895
Bone marrow involvement	0.100	0.167
Impact of antiviral treatment		
Overall survival	0.917	0.407
Progression-free survival/disease-free survival	0.260	0.070
Liver-related survival factors		
Liver cirrhosis	0.707	0.262

**Abbreviation: ***H-I/H* high-intermediate and high

## Discussion

Over the past two decades, considerable studies have investigated the contribution of HCV infection to lymphomagenesis. However, the mechanisms of HCV-associated lymphoma remain elusive. Experimental data showed that HCV-induced lymphoma development may act through multistep processes involving a variety of oncogenic mechanisms, such as HCV-induced chronic B-cell immune stimulation, genetic damage, and dysregulation of signalling pathways (NF-kB, JNF, ERK and NOTCH signalling pathways) [43–48]. A prognostic evaluation of clinical outcomes and therapeutic responses in HCV-associated lymphoma is essential to antitumour treatment. Herein, we performed a systematic review and meta-analysis to assess the impact of HCV infection on NHL patients. Furthermore, subgroup analysis was also performed based on the prevalence of HCV infection and the pathological types of lymphoma. The results of the current study collectively indicated that HCV-positive NHL patients had significantly inferior survivals, earlier disease progression, worse treatment responses and distinct clinical characteristics. In addition to the recognized survival factors of NHL, our study also demonstrated that liver cirrhosis and low levels of albumin were inferior prognostic factors of OS for HCV-associated NHL patients.

Furthermore, this study implicated the anti-lymphoma efficacy of antiviral treatment, especially IFN ± ribavirin regimens, in HCV-associated NHL patients, which was similar to the findings of a recent meta-analysis conducted by Peveling-Oberhag et al. [49]. The authors found a good overall lymphoma response rate through the application of antiviral treatment in HCV-infected NHL patients. However, their meta-analysis was based on small studies, mostly case reports or small cohorts with fewer than 20 cases. Nevertheless, DAA treatments are radically changing the landscape of anti-HCV therapy. In the current study, whether HCV-associated NHL patients can benefit from DAA treatment could not be expounded upon due to the small patient sample size. In the era of DAA, future studies should investigate the anti-lymphoma activity of DAA treatment in NHL patients with HCV infection.

On the other hand, rituximab-containing regimens have been proven to be associated with better treatment responses and clinical prognosis in DLBCL over the past two decades. In the current study, we also found that rituximab administration could improve OS in DLBCL patients with HCV infection. However, the association between PFS and rituximab administration could not be observed in this specific subtype of DLBCL patients, which might be due to the small sample size. Whether rituximab-containing regimens can improve the treatment response and PFS warrants further study with larger cohorts.

To the best of our knowledge, this is the first meta‐analysis that systematically assessed the clinical outcomes and treatment of HCV-associated NHL patients. We are confident that our results are reliable, since they were supported by a large sample size, moderate to high methodological quality of the included studies, low heterogeneity, and no publication bias. However, there were several limitations of this study. First, when evaluating OS or PFS/DFS, HRs and 95% CIs in some individual studies were not available from the original articles; hence, they were indirectly calculated from Kaplan–Meier curves. Second, our study found that a higher incidence of hepatic dysfunction was observed in HCV-infected NHL patients, probably leading to delays or termination of chemotherapy or even death. However, the direct cause-and-effect relationship between hepatic dysfunction and the inferior prognosis remains unknown. On the other hand, it is still unclear whether the survival benefit of antiviral treatment is attributed to restoring hepatic dysfunction and reducing the chance of termination or delay of chemotherapy caused by HCV-induced hepatotoxicity. Third, our study found that HCV infection was associated with poor survival in NHL patients. However, when we attempted to further perform prognostic evaluation of HCV viral loading (HCV-RNA) in HCV-infected NHL patients, we found that only two articles investigated the prognostic value of HCV-RNA, and the results were contradictory. Fourth, it should be noted that when analysing some outcomes, such as prognostic factors and the effect of rituximab administration to HCV-associated NHL patients, only a few studies with small sample sizes could be included. Therefore, the results of these outcomes need to be interpreted with caution. Fifth, although we performed comprehensive literature search, most of the included studies were retrospective studies. Therefore, HCV-negative NHL patients in our study might not fully represent the whole HCV-negative NHL population. Last, patients in most of the included studies had DLBCL. There were only a few studies focusing on indolent lymphoma, such as follicular lymphoma and marginal zone lymphoma; hence, subgroup analysis could not be carried out for indolent lymphoma patients. In view of the limitations of the current study, well-designed prospective studies with large cohorts should be further performed in the different subtypes of NHL, especially indolent lymphoma, to address the issues mentioned above.

## Conclusions

This meta-analysis confirmed the inferior prognosis and distinct clinical characteristics of HCV-associated NHL patients, especially in DLBCL. Patients with HCV infection were prone to undergoing hepatic dysfunction during chemotherapy. Moreover, our data provide compelling evidence that antiviral treatment combined with immunochemotherapy may represent an effective approach for HCV-positive NHL patients.

## Supplementary Information

**Additional file 1: Table S1.** Literature search strategy in different electronic databases.

**Additional file 2: Table S2.** Quality assessment results of included studies.

**Additional file 3: Figure S1.** Meta-analysis of the association between HCV status and advanced disease stage (Ann Arbor staging III/IV) in NHL patients.

**Additional file 4: Figure S2.** Meta-analysis of the association between HCV status and elevated LDH levels in NHL patients.

**Additional file 5: Figure S3.** Meta-analysis of the association between HCV status and the intermediate-high and high IPI/FLIPI risk in NHL patients.

**Additional file 6: Figure S4.** Meta-analysis of the association between HCV status and spleen involvement in NHL patients.

**Additional file 7: Figure S5.** Meta-analysis of the association between HCV status and liver involvement in NHL patients.

**Additional file 8: Figure S6.** Meta-analysis of the association between HCV status and the age of disease onset in NHL patients.

**Additional file 9: Figure S7.** Meta-analysis of the association between HCV status and the presence of B symptoms in NHL patients.

**Additional file 10: Figure S8.** Meta-analysis of the association between HCV status and bone marrow involvement in NHL patients.

## Data Availability

The datasets used in this study are available from the corresponding author upon reasonable request.

## Abbreviations

ALT: Alanine transaminase
CI: Confidence interval
DAA: Direct-acting antiviral
DFS: Disease-free survival
DLBCL: Diffuse large B-cell lymphoma
HCV: Hepatitis C virus
HR: Hazard ratio
IFN: Interferon
NHL: Non-Hodgkin’s lymphoma
NOS: Newcastle–Ottawa Quality Assessment Scale
PFS: Progression-free survival
OR: Odds ratio
ORR: Overall response rate
OS: Overall survival
WHO: World Health Organization
