# Supplementary material for: Distinct clinical features and prognostic factors of hepatitis C virus-associated non-Hodgkin’s lymphoma: a systematic review and meta-analysis
Source: Cancer Cell Int. 2021 Oct 9;21:524. doi: 10.1186/s12935-021-02230-1 (PMC8502277; doi:10.1186/s12935-021-02230-1)
Supplement: Supplementary file 1 — Additional file 1: Table S1. Literature search strategy in different electronic databases. [file 12935_2021_2230_MOESM1_ESM.docx]

| Databases | Period | Search strategy |
| --- | --- | --- |
| PubMed | ~Jul 31, 2021 | #1 “Lymphoma, Non-Hodgkin” [Mesh]  #2 “non-Hodgkin’s lymphoma”  #3 “NHL”  #4 “Hepatitis C” [Mesh]  #5 “[Hepatitis C, Chronic](https://www-ncbi-nlm-nih-gov.eproxy.lib.hku.hk/mesh/68019698)” [Mesh]  #6 “[Hepatitis C Antibodies](https://www-ncbi-nlm-nih-gov.eproxy.lib.hku.hk/mesh/68018937)” [Mesh]  #7 “[Hepatitis C Antigens](https://www-ncbi-nlm-nih-gov.eproxy.lib.hku.hk/mesh/68018936)” [Mesh]  #8 “hepatitis C”  #9 “hepatitis C virus”  #10 “HCV”  #11 “Hep C virus”  #12 “Hep C”  #13 #1 OR #2 OR #3  #14 #4 OR #5 OR #6 OR #7 OR #8 OR #9 OR #10 OR #11 OR #12  #15 #13 AND #14 |
| EMBASE/OVID | ~Jul 31, 2021 | #1 exp non-Hodgkin's lymphoma/  #2 non-Hodgkin's lymphoma.mp. or nonhodgkin lymphoma/  #3 nonhodgkin lymphoma/ or NHL.mp.  #4 exp hepatitis c/  #5 exp Hepatitis C virus/  #6 exp hepatitis c, chronic/  #7 HCV.mp. or hepatitis C/  #8 Hepatitis C/ or Hepatitis c.mp.  #9 Hepatitis C virus.mp. or Hepatitis C virus/  #10 #1 OR #2 OR #3  #11 #4 OR #5 OR #6 OR #7 OR #8 OR #9  #12 #10 AND #11 |
| The Cochrane Central Register of Controlled Trials (CENTRAL/CCTR) | ~Jul 31, 2021 | #1 MeSH descriptor: [Lymphoma, Non-Hodgkin] explode all trees  #2 MeSH descriptor: [Hepatitis C] explode all trees  #3 MeSH descriptor: [Hepatitis C virus] explode all trees  #4 “non-hodgkin’s lymphoma” or “NHL” :ti,ab,kw (Word variations have been searched)  #5 “hcv” or "hepatitis c" or "hepatitis c virus":ti,ab,kw (Word variations have been searched)  #6 #1 OR #4  #7 #2 OR #3 OR #5  #8 #6 AND #7 |
| ClinicalTrials | ~Jul 31, 2021 | #1 “Lymphoma, Non-Hodgkin” AND “Hepatitis C”  #2 “Lymphoma, Non-Hodgkin” AND “[Hepatitis C, Chronic](https://www-ncbi-nlm-nih-gov.eproxy.lib.hku.hk/mesh/68019698)”  #3 “Lymphoma, Non-Hodgkin” AND “hepatitis C virus”  #4 “Lymphoma, Non-Hodgkin” AND “hepatitis C virus infection”  #5 “Lymphoma, Non-Hodgkin” AND “HCV”  #6 “NHL AND “Hepatitis C”  #7 “NHL” AND “[Hepatitis C, Chronic](https://www-ncbi-nlm-nih-gov.eproxy.lib.hku.hk/mesh/68019698)”  #8 “NHL” AND “hepatitis C virus”  #9 “NHL” AND “hepatitis C virus infection”  #10 “NHL” AND “HCV” |
