# Supplementary figures and images for: Distinct clinical features and prognostic factors of hepatitis C virus-associated non-Hodgkin’s lymphoma: a systematic review and meta-analysis
Source: Cancer Cell Int. 2021 Oct 9;21:524. doi: 10.1186/s12935-021-02230-1 (PMC8502277; doi:10.1186/s12935-021-02230-1)

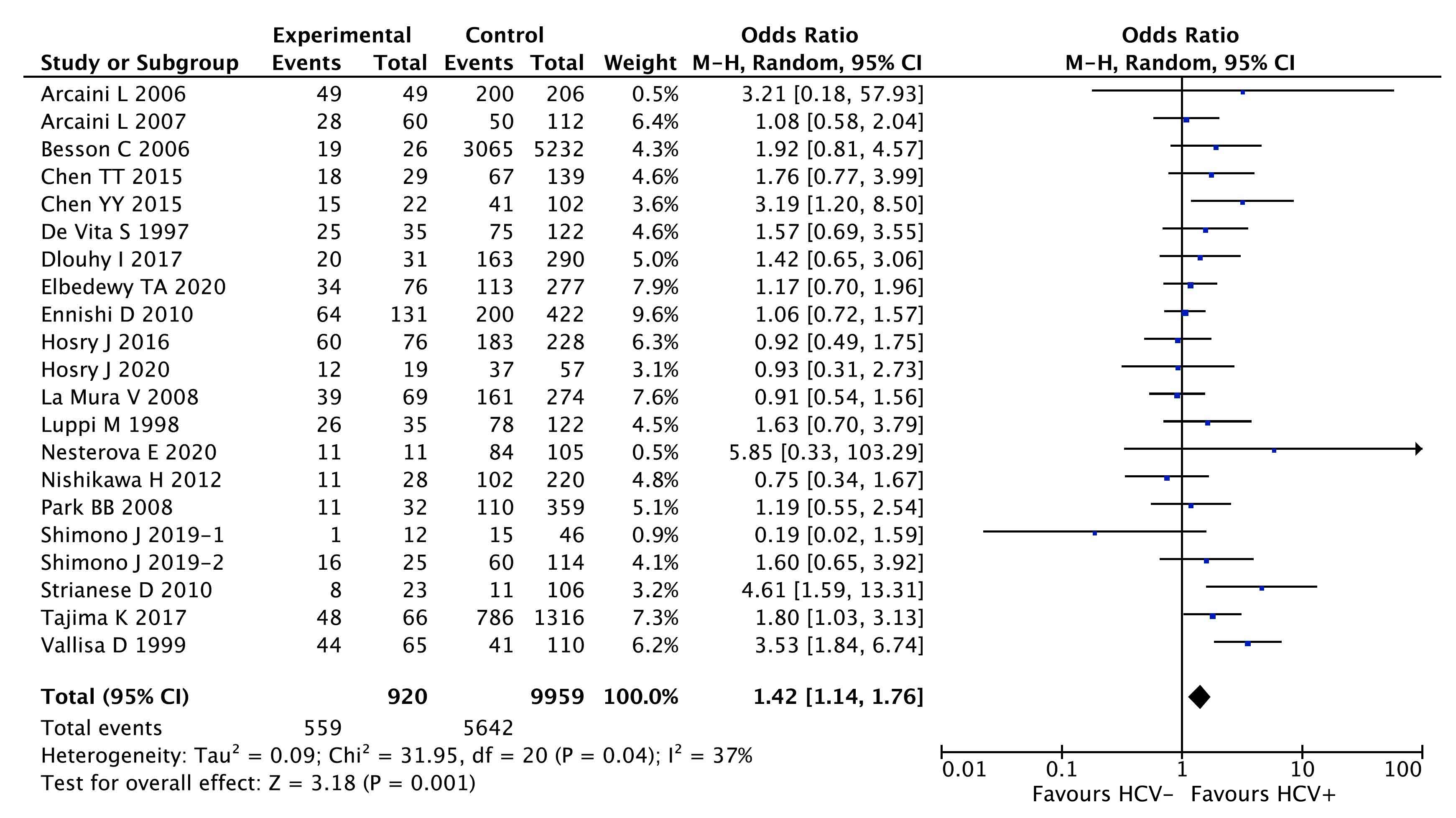

Supplement: Supplementary file 3 — Additional file 3: Figure S1. Meta-analysis of the association between HCV status and advanced disease stage (Ann Arbor staging III/IV) in NHL patients. [file 12935_2021_2230_MOESM3_ESM.jpg]

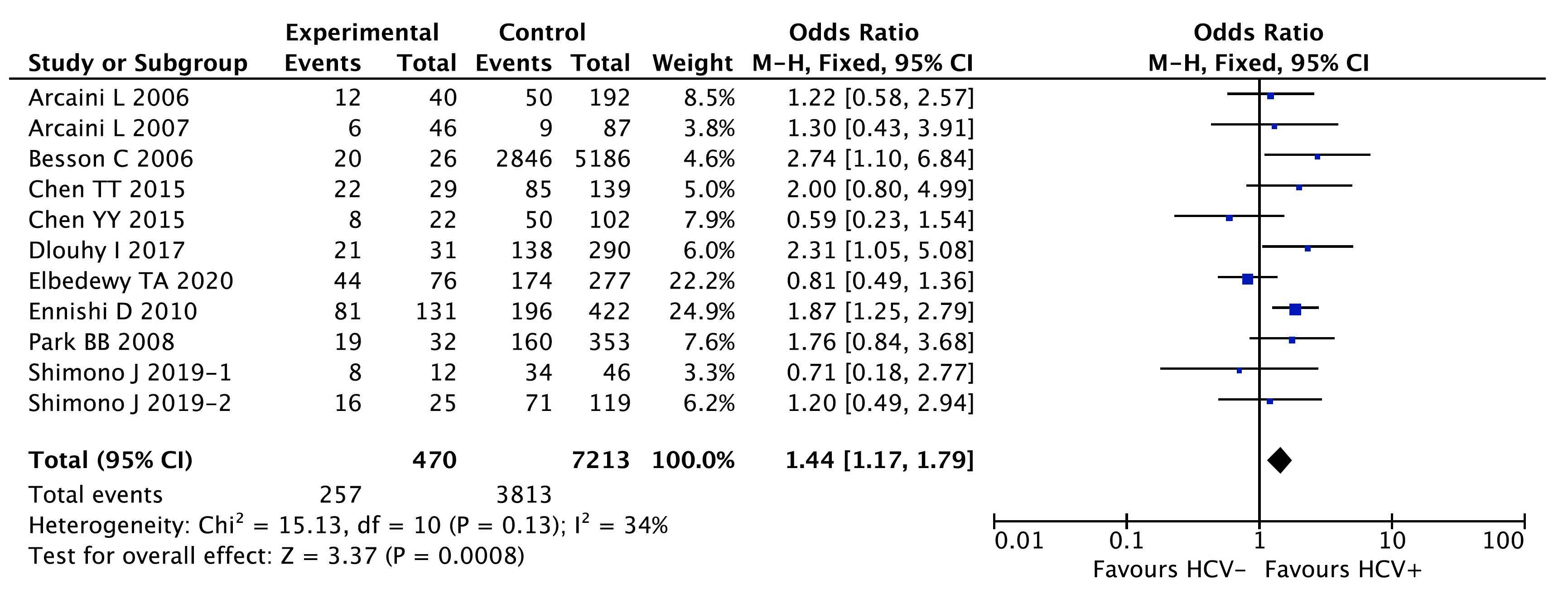

Supplement: Supplementary file 4 — Additional file 4: Figure S2. Meta-analysis of the association between HCV status and elevated LDH levels in NHL patients. [file 12935_2021_2230_MOESM4_ESM.jpg]

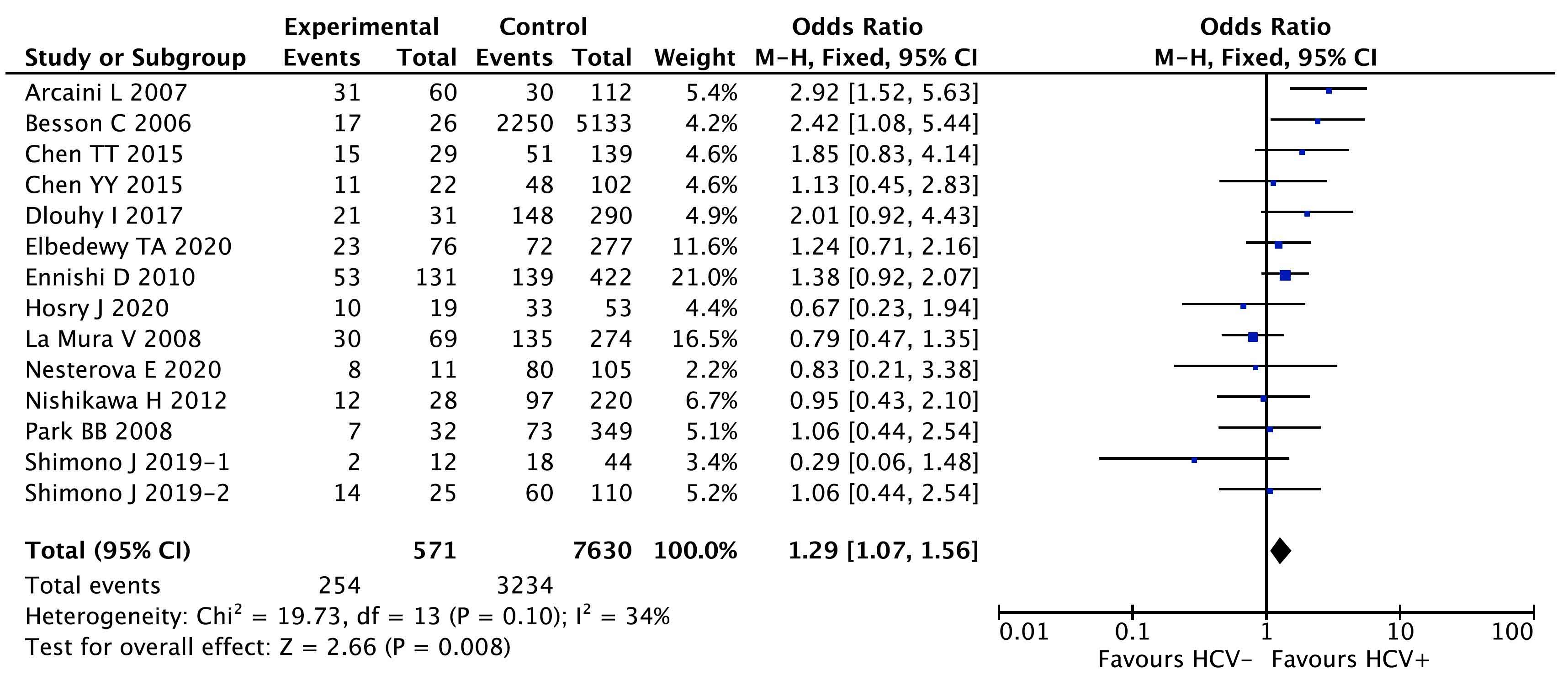

Supplement: Supplementary file 5 — Additional file 5: Figure S3. Meta-analysis of the association between HCV status and the intermediate-high and high IPI/FLIPI risk in NHL patients. [file 12935_2021_2230_MOESM5_ESM.jpg]

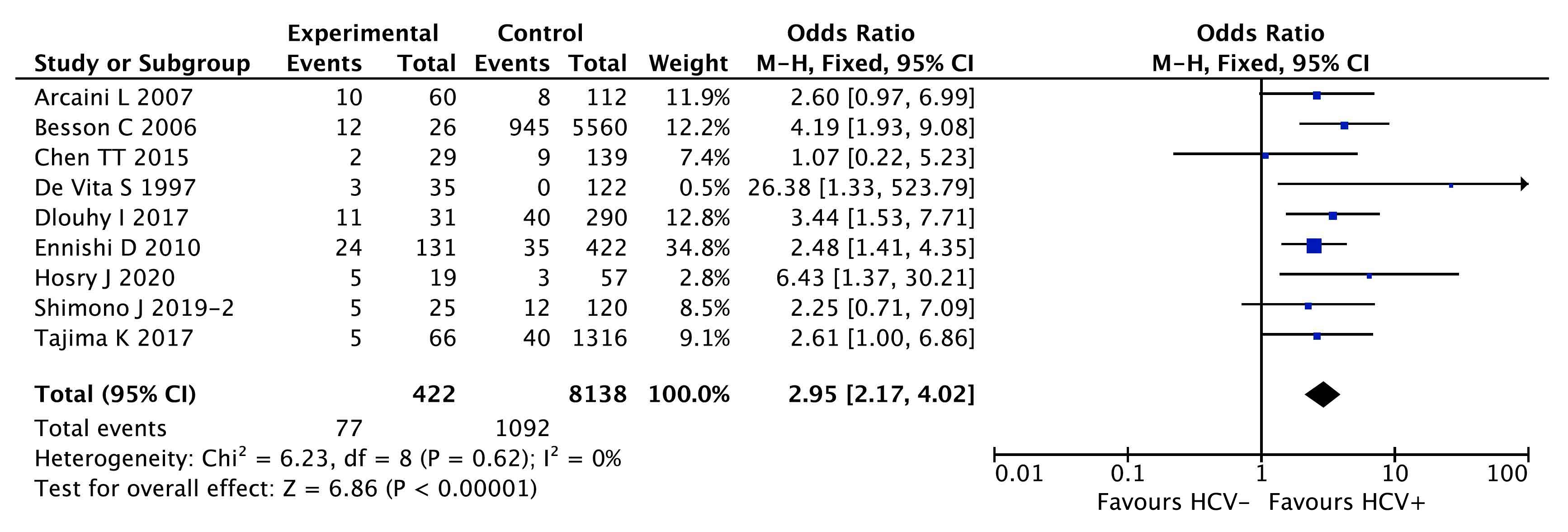

Supplement: Supplementary file 6 — Additional file 6: Figure S4. Meta-analysis of the association between HCV status and spleen involvement in NHL patients. [file 12935_2021_2230_MOESM6_ESM.jpg]

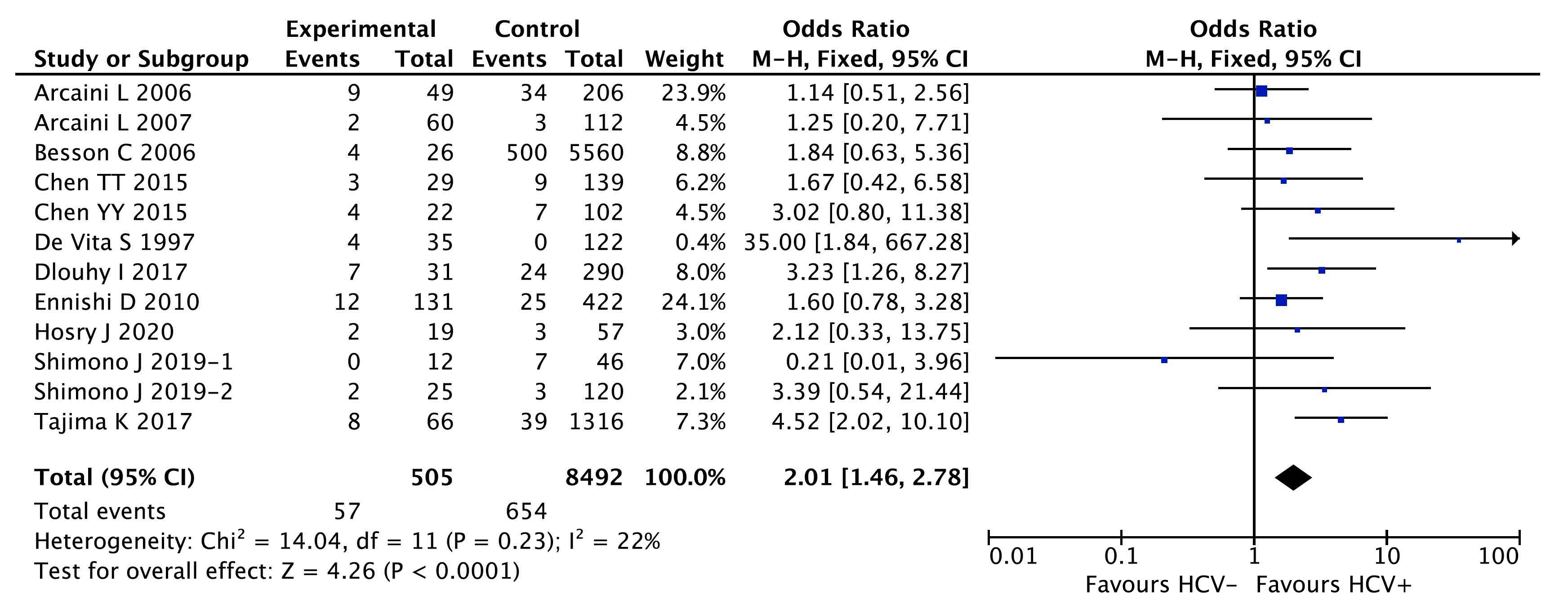

Supplement: Supplementary file 7 — Additional file 7: Figure S5. Meta-analysis of the association between HCV status and liver involvement in NHL patients. [file 12935_2021_2230_MOESM7_ESM.jpg]

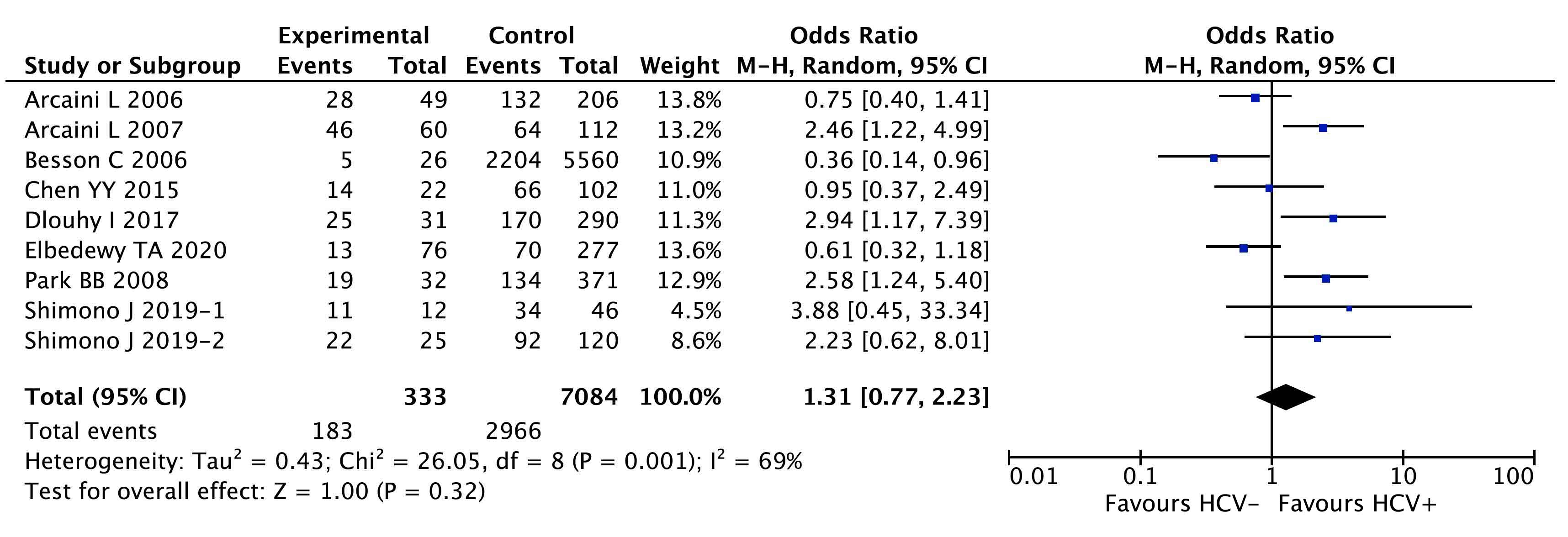

Supplement: Supplementary file 8 — Additional file 8: Figure S6. Meta-analysis of the association between HCV status and the age of disease onset in NHL patients. [file 12935_2021_2230_MOESM8_ESM.jpg]

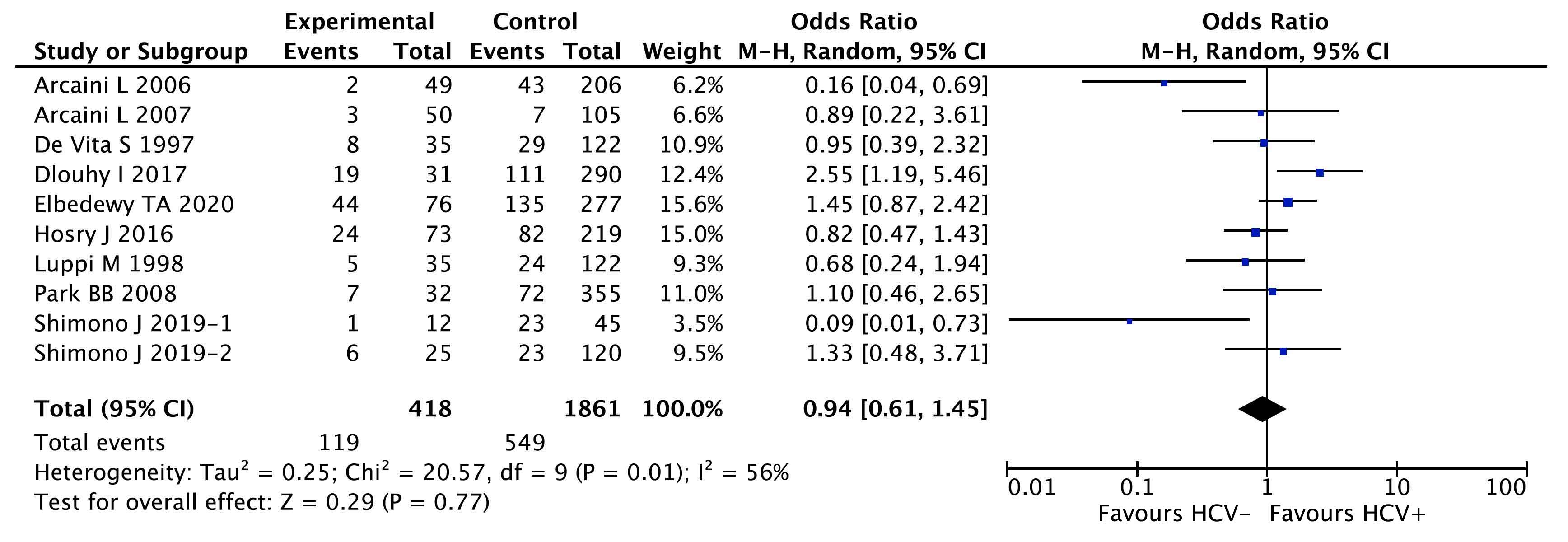

Supplement: Supplementary file 9 — Additional file 9: Figure S7. Meta-analysis of the association between HCV status and the presence of B symptoms in NHL patients. [file 12935_2021_2230_MOESM9_ESM.jpg]

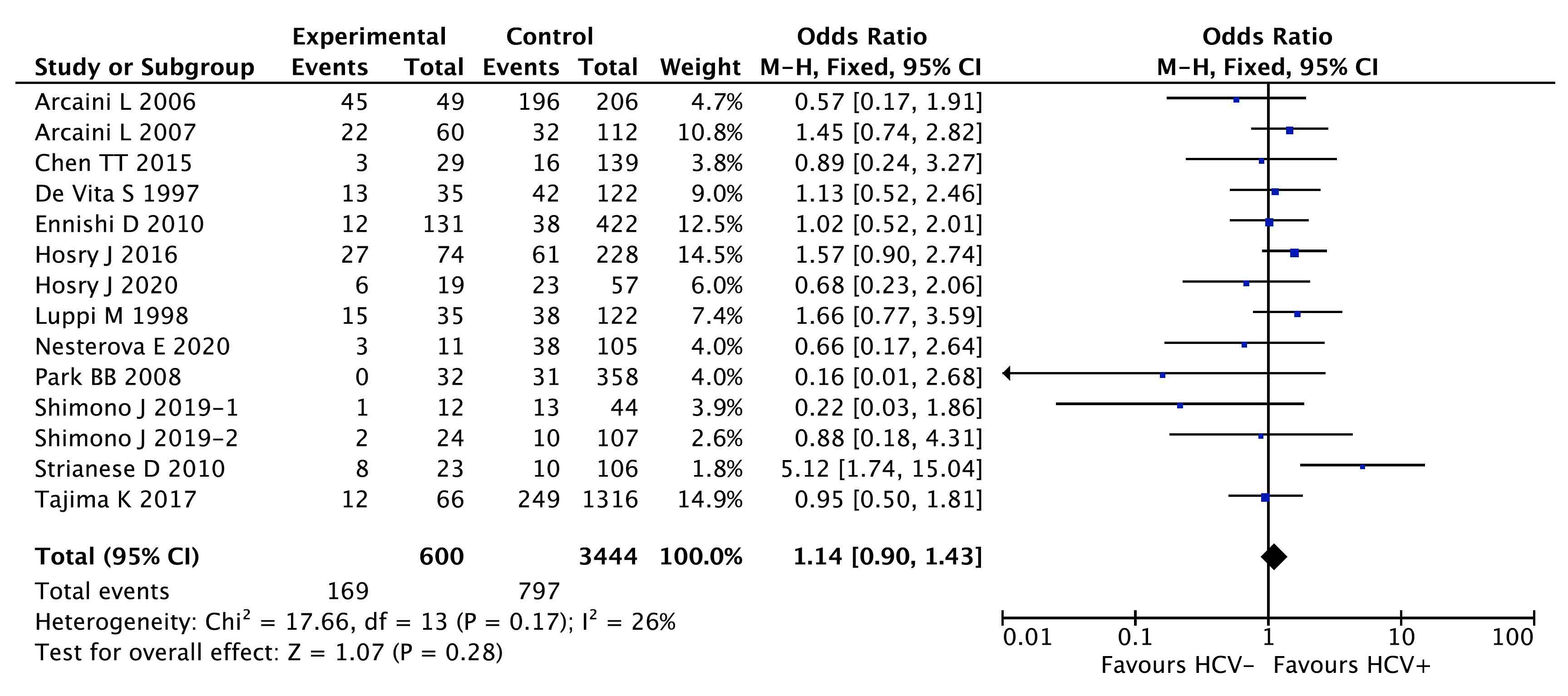

Supplement: Supplementary file 10 — Additional file 10: Figure S8. Meta-analysis of the association between HCV status and bone marrow involvement in NHL patients. [file 12935_2021_2230_MOESM10_ESM.jpg]
